# Influence of Hydrolysis Medium on Matrix Degradation and Carbon Fibre Recovery from Epoxy-Based CFRPs

**DOI:** 10.3390/polym18202458

**Published:** 2026-10-09

**Authors:** Alen Erjavec, Tjan Budja, Kenan Kapetanović, Sara Perša, Michela Sossi, Silvo Hribernik, Julija Volmajer Valh

**Affiliations:** 1 Faculty of Mechanical Engineering, University of Maribor, Smetanova ulica 17, 2000 Maribor, Slovenia; alen.erjavec@um.si (A.E.); tjan.budja@student.um.si (T.B.); kenan.kapetanovic@student.um.si (K.K.); sara.persa@student.um.si (S.P.);; 2 Faculty of Electrical Engineering and Computer Science, University of Maribor, Koroška cesta 46, 2000 Maribor, Slovenia; silvo.hribernik@um.si

**Keywords:** carbon fibre-reinforced polymers (CFRPs), chemical recycling, alkaline hydrolysis, fibre recovery, thermoset composites

## Abstract

The increasing use of carbon fibre-reinforced polymers (CFRPs) has intensified the need for efficient recycling technologies capable of recovering high-value carbon fibres from end-of-life composites. This study compares neutral and alkaline hydrolysis for recycling epoxy-based CFRPs under identical conditions (250 °C, 37–40 bar). CFRP waste generated during the manufacture of Formula Student race car components was treated in deionised water or 1.04 M NaOH for 60 or 120 min. Matrix degradation and fibre recovery were evaluated by gravimetric analysis, pH and conductivity measurements, Fourier transform infrared spectroscopy (FTIR), scanning electron microscopy (SEM), and X-ray diffraction (XRD). Neutral hydrolysis resulted in only 4.5% mass loss and limited matrix degradation. In contrast, alkaline hydrolysis achieved mass losses of 34.2% and 38.7% after 60 and 120 min, respectively, with more extensive matrix removal observed after the longer treatment. Under strongly alkaline conditions, hydroxide ions can promote the cleavage of susceptible bonds within the epoxy network, facilitating matrix depolymerisation and removal. After 60 min, partial matrix removal was observed, whereas 120 min resulted in nearly complete matrix removal and successful carbon fibres recovery. FTIR, SEM, and XRD confirmed effective matrix removal while preserving fibre morphology and crystalline structure. The results demonstrate that alkaline hydrolysis is significantly more effective than neutral hydrolysis for recycling epoxy-based CFRPs and highlight the importance of combining complementary analytical techniques to reliably evaluate recycling efficiency.

## 1. Introduction

Carbon fibre-reinforced polymers (CFRPs) are among the most important high-performance composite materials used in modern engineering applications. Their exceptional specific strength, stiffness, fatigue resistance, and corrosion resistance have enabled widespread adoption in aerospace, automotive, wind energy, marine, and sporting industries. The continuous demand for lightweight structures and energy-efficient transportation systems has accelerated CFRP production worldwide, resulting in a parallel increase in manufacturing scrap and end-of-life composite waste. As CFRP consumption continues to expand, sustainable management of composite waste has become a critical technological and environmental challenge [1,2,3].

The recycling of CFRPs is considerably more complex than that of conventional metallic materials because of their heterogeneous structure, consisting of high-value carbon fibres embedded within a polymer matrix. In high-performance applications, epoxy resins are the most commonly employed matrix materials due to their excellent adhesion, thermal stability, chemical resistance, and mechanical performance. However, the highly crosslinked three-dimensional network structure of cured epoxy resins prevents remelting and reshaping after use, making recycling significantly more challenging than for thermoplastic composites [4,5].

Several recycling technologies have been developed to address the growing volume of CFRP waste. Mechanical recycling, based on shredding, grinding, or milling, represents the simplest and most economical approach. However, the process inevitably shortens carbon fibres and reduces their reinforcing capability, limiting the value of the recovered material. Thermal recycling methods, particularly pyrolysis and fluidised-bed processing, can effectively remove the polymer matrix and recover carbon fibres, but high temperatures may alter fibre surface chemistry, reduce interfacial performance, and increase energy consumption [2,6].

Chemical recycling has emerged as one of the most promising routes for the recovery of high-quality carbon fibres from thermoset composites. Unlike mechanical and thermal approaches, chemical processes selectively depolymerise the polymer matrix through bond cleavage while minimizing structural damage to the reinforcing fibres. Various chemical recycling techniques have been investigated, including solvolysis, glycolysis, alcoholysis, hydrolysis, and supercritical fluid treatment [7,8]. Depending on the process conditions, chemical recycling can achieve near-complete matrix degradation while preserving a substantial proportion of the original fibre properties [2,9].

Among chemical recycling approaches, hydrolysis has attracted increasing attention because it utilises water-based reaction systems that are generally more environmentally acceptable than many organic solvent systems. Under elevated temperature and pressure, water penetrates the crosslinked epoxy network and promotes cleavage of hydrolytically susceptible bonds. However, the efficiency of neutral hydrolysis is often limited by slow reaction kinetics and incomplete degradation of highly crosslinked thermoset structures [9].

To enhance resin degradation, alkaline catalysts are frequently introduced into the reaction medium. Hydroxide ions can promote the cleavage of susceptible bonds within the epoxy network, accelerating depolymerisation and dissolution of matrix fragments. Previous studies have shown that alkaline environments can promote hydrolysis of epoxy resins, leading to changes in the molecular structure and to microstructural deterioration, including the formation of micropores and microcracks [10,11]. At the composite level, alkaline exposure has also been associated with degradation of the epoxy matrix and fibre–matrix interface, resulting in matrix loss, pore formation, and deterioration of mechanical performance [12,13]. Previous studies have demonstrated that alkaline-assisted recycling significantly improves matrix removal compared with non-catalytic hydrolysis and can enable recovery of carbon fibres with minimal structural damage [14,15]. Furthermore, analyses based on scanning electron microscopy (SEM), X-ray diffraction (XRD), and mechanical testing have shown that the characteristic crystalline structure and mechanical performance of recovered fibres can be largely pre-served when process conditions are properly controlled [5,9,16].

Recent developments in chemical recycling have focused on improving process efficiency, reducing energy consumption, and recovering both carbon fibres and value-added degradation products from the epoxy matrix. Selective depolymerisation has emerged as one of the most promising strategies for the sustainable recycling of thermoset composites, enabling simultaneous recovery of high-quality carbon fibres and value-added chemical products while supporting circular economy principles [17]. Solvolytic and catalytic recycling systems have achieved resin degradation efficiencies exceeding 90–99% while maintaining a high proportion of the original fibre strength. Nevertheless, process performance remains highly dependent on operating parameters, including reaction medium composition, catalyst concentration, temperature, pressure, and treatment duration [16,18,19]. 

Despite significant progress in recent years, direct comparisons between neutral and alkaline hydrolysis performed under identical reaction conditions remain limited. Moreover, many studies primarily focus on resin removal efficiency, whereas fewer investigations combine complementary analytical techniques to evaluate both matrix degradation and preservation of fibre structure. A comprehensive assessment employing spectroscopic, microscopic, and structural characterisation is therefore required to better understand the effectiveness of hydrolysis-based recycling strategies for epoxy-based CFRPs, targeting both resin removal and fibre recuperation.

The novelty of this study lies in the direct comparison of neutral and alkaline hydrolysis under identical temperature and pressure conditions, combined with complementary analytical techniques to evaluate both matrix degradation and carbon fibre recovery.

Therefore, the aim of this study was to systematically compare neutral and alkaline hydrolysis for the recycling of epoxy-based CFRP waste generated during the manufacture of Formula Student race car components by the University of Maribor GPE Team. Neutral hydrolysis in deionised water and alkaline hydrolysis in sodium hydroxide solution was performed under identical temperature and pressure conditions in a high-pressure reactor. The extent of matrix degradation and fibre recovery was evaluated using gravimetric analysis, pH and conductivity measurements, Fourier transform infrared spectroscopy (FTIR), scanning electron microscopy (SEM), and X-ray diffraction (XRD). Particular attention was devoted to evaluating the influence of reaction medium and treatment duration on epoxy matrix degradation, fibre recovery efficiency, and the preservation of the morphology and structural integrity of the recovered carbon fibres.

## 2. Materials and Methods

### 2.1. Materials

**Figure 1:**
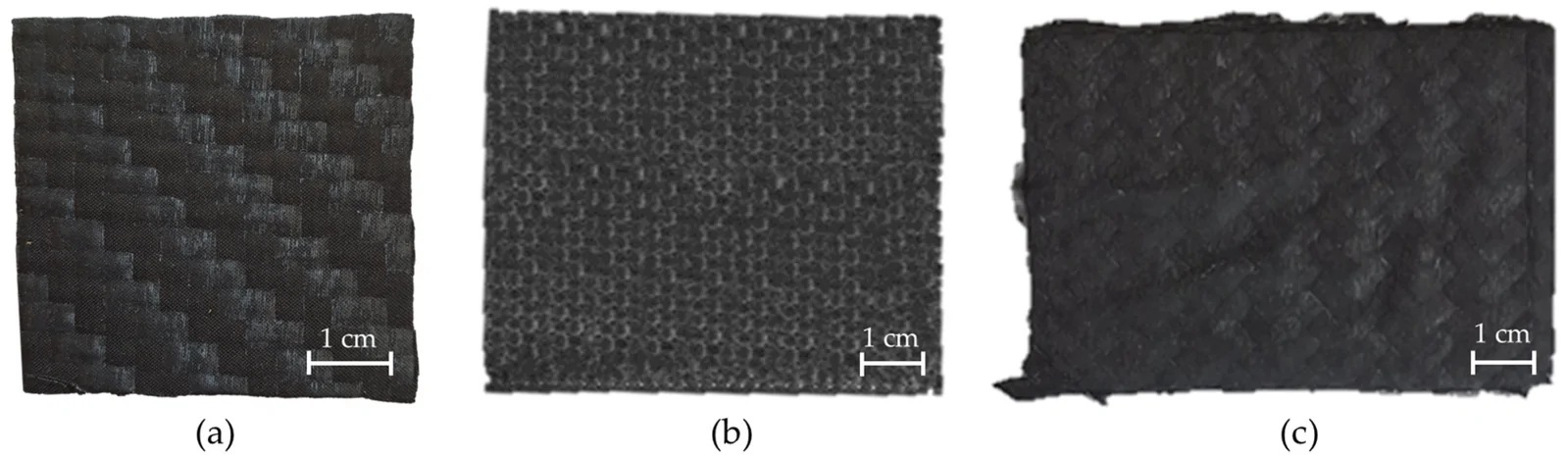
CFRP samples: (**a**) sample 1, (**b**) sample 2 and (**c**) sample 3.

Three epoxy-based CFRP specimens were selected for the experimental investigation. All specimens were obtained as production waste from the manufacture of Formula Student race car components by the University of Maribor–GPE Team and were therefore not specifically prepared to have identical dimensions and mass. Samples 1 and 2 had comparable dimensions and mass allowing for a direct comparison of their behaviour under different hydrolysis conditions. In contrast, Sample 3 was characterised by a significantly greater mass and laminate thickness (Figure 1). The CFRP material consisted of carbon fibres embedded in a DT120 thermosetting epoxy resin matrix. According to the manufacturer’s technical data sheet, the resin content of DT120 carbon fibre fabric prepregs ranges from 37 to 42 wt.%, corresponding to a carbon fibre content of approximately 58–63 wt.%, depending on the carbon fibre fabric used. The exact initial matrix and carbon fibre mass fractions of the individual production waste specimens were not experimentally determined.

### 2.2. Methods

#### 2.2.1. Hydrolysis

The hydrolysis experiments were conducted in a 1 L stainless steel high-pressure reactor (Ecom, Ruše, Slovenia). Three CFRP specimens were processed under controlled laboratory conditions to evaluate the effects of neutral and alkaline hydrolysis on matrix removal and fibre recovery. In all experiments, a constant liquid volume of 250 mL was used, and the temperature was maintained at 250 °C. The corresponding autogenous pressure inside the reactor ranged between 37 and 40 bar. The reaction time was measured only after the system reached steady-state temperature and pressure. Due to the exploratory nature of the study and limitations associated with reactor availability, each hydrolysis condition was evaluated using a single representative CFRP specimen. Therefore, the results should be interpreted as a comparative proof-of-concept rather than a statistical evaluation. Employed reactor set-up also resulted in subsequent inability to perform reliable mechanical testing of the recovered fibres; their length after recovery was insufficient for tensile characterisation. The use of larger CFRP specimens, which could have yielded longer fibre segments, was constrained by the dimensions and working volume of the high-pressure reactor. The reported mass loss was calculated from the difference between the initial specimen mass and the recovered dried solid mass. Since dissolved or dispersed degradation products in the liquid phase were not quantitatively recovered, a complete mass balance could not be established.

#### 2.2.2. Neutral Hydrolysis

Sample 1 (1.5975 g) was subjected to neutral hydrolysis in 250 mL of deionised water for 60 min at 250 °C. After completion, the liquid and solid phases were separated. The solid residues were dried and weighed using an ALT 220-4NM analytical balance (Kern, Balingen, Germany) with a precision of ±0.001 g prior to further analysis.

#### 2.2.3. Alkaline Hydrolysis

Samples 2 and 3 were treated under identical temperature and pressure conditions, using a 1.04 M sodium hydroxide (NaOH) aqueous solution as the reaction medium. The NaOH solution was prepared using deionised water and NaOH mini pellets (Fluka, Buchs, Switzerland). Sample 2 (1.1577 g) was exposed for 60 min, while Sample 3 (9.6146 g) underwent 120 min of alkaline hydrolysis. At the end of each process, the liquid phase was filtered, and the solid residue was thoroughly washed with distilled water, dried, and weighed prior to characterisation. 

#### 2.2.4. Vacuum Filtration

Following hydrolysis, the separation of the liquid and solid phases was performed by vacuum filtration using a water jet vacuum pump and cellulose filter papers. This step enabled efficient removal of residual solution and collection of fine particulate material for further analysis. The filtrates were stored in sealed glass containers, while the solid residues were washed thoroughly with distilled water before drying.

#### 2.2.5. Drying Procedure

The solid residues were dried at 105 °C in a Sterimatic ST-11 drying oven (Instrumen-taria, Zagreb, Croatia) to remove residual moisture and volatile compounds that could interfere with subsequent spectroscopic and microscopic analyses.

#### 2.2.6. Analytical Methods

A series of analytical techniques were employed to characterise the liquid and solid phases after hydrolysis and to assess the extent of matrix removal and fibre recovery.

#### 2.2.7. pH and Conductivity Measurements

The pH and conductivity of the liquid phase samples were measured using a SevenMulti™ multiparameter meter (Mettler Toledo, Greifensee, Switzerland) equipped with a conductivity and a combined pH/temperature electrode. The measurement accuracy was ±0.002 for pH and ±0.5% of the reading for conductivity. Both parameters were determined for the liquid phase of all three samples before and after hydrolysis to monitor chemical changes and evaluate the extent of polymer depolymerisation.

#### 2.2.8. Fourier Transform Infrared Spectroscopy (FTIR)

ATR-FTIR spectra were recorded using a Perkin Elmer Spectrum GX spectrometer (PerkinElmer, Waltham, MA, USA) equipped with a diamond ATR crystal (Specac Ltd., Orpington, UK). For each sample, 16 scans were collected at a resolution of 4 cm^−1^, with a penetration depth of approximately 0.75 mm. Spectra were acquired at ambient temperature over the wavenumber range of 4000–650 cm^−1^ to identify characteristic functional groups and monitor matrix decomposition.

#### 2.2.9. X-Ray Diffraction (XRD)

The crystalline structure of the hydrolysed samples was determined using a Bruker D2 Phaser powder diffractometer (Bruker AXS, Karlsruhe, Germany) with Cu–Kα radiation (λ = 1.5406 Å). X-ray diffraction patterns were collected in the 2θ range from 5° to 70°, with a step size of 0.03° and a counting time of 0.2 s per step. Cut specimens (10 mm × 10 mm × 4 mm) were mounted on a zero-background silicon sample holder. The degree of crystallinity was determined using the diffrac. EVA software package (Bruker, Karlsruhe, Germany; version 5.1.0.5).

#### 2.2.10. Scanning Electron Microscopy (SEM)

Morphological characterisation of the recovered carbon fibres and the composite fracture surfaces was performed using a Carl Zeiss FE-SEM SUPRA 35 VP scanning electron microscope (Carl Zeiss, Jena, Germany) equipped with a GEMINI field emission module. High-resolution micrographs were obtained to evaluate the surface morphology, fibre–matrix interface, and potential structural damage to the carbon fibres after hydrolysis.

## 3. Results and Discussion

### 3.1. Characterisation of the Polymer Matrix

First, the chemical composition of the composite matrix with particular focus on identifying the polymer present alongside the carbon fibres, was performed. Therefore, FTIR spectra were recorded for each sample.

**Figure 2:**
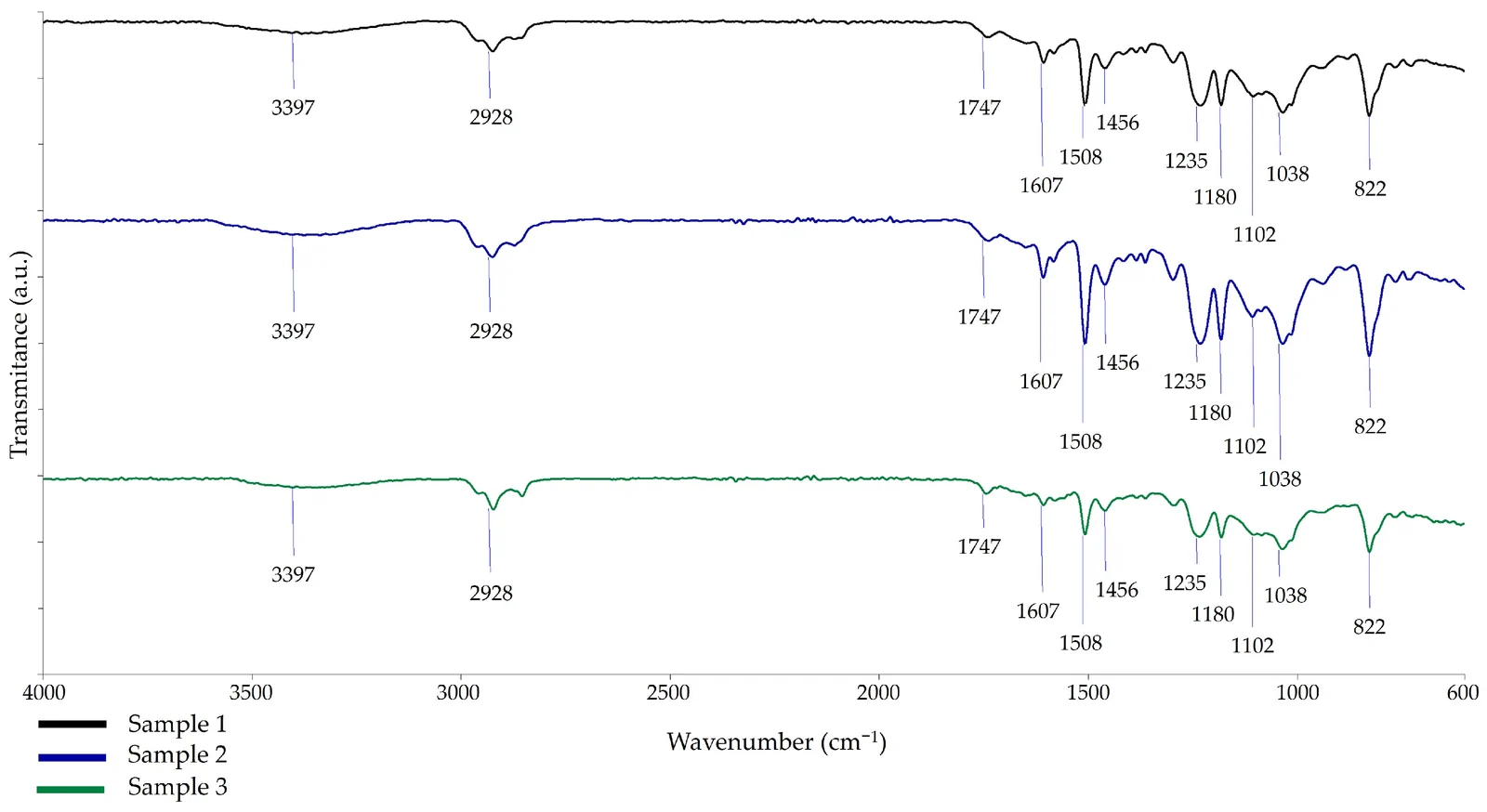
FTIR spectrum of CFRP samples.

The FTIR spectra of sample 1, sample 2 and sample 3 (Figure 2) show characteristic signals of a cured epoxy matrix. A broad signal at approximately 3397 cm^−1^ is attributed to O–H stretching vibrations originating from hydroxyl groups formed during the epoxy curing reaction. The signal at 2928 cm^−1^ corresponds to aliphatic C–H stretching vibrations. Characteristic aromatic skeletal vibrations of the bisphenol-A-based epoxy network are observed at 1607 and 1508 cm^−1^, while the signal at 1456 cm^−1^ is assigned to CH_2_ bending vibrations. Strong absorption signals at 1235, 1180, 1102, and 1038 cm^−1^ are associated with C–O–C stretching vibrations of ether linkages within the crosslinked epoxy structure. The signal at 822 cm^−1^ corresponds to out-of-plane aromatic C–H bending vibrations of para-substituted benzene rings. The absence of the characteristic epoxy ring vibration at approximately 915 cm^−1^ confirms the consumption of epoxide groups during curing and indicates the formation of a fully crosslinked epoxy network. The signal observed at 1747 cm^−1^ is attributed to carbonyl (C=O) stretching vibrations. This signal may originate from ester-containing toughening agents or other proprietary modifiers incorporated into the epoxy system to enhance fracture toughness [20].

Epoxy resins are thermosetting polymers formed by the reaction of epoxide-containing monomers with suitable curing agents. During curing, a highly crosslinked three-dimensional network is formed, resulting in excellent mechanical properties, dimensional stability, and chemical resistance. Owing to their versatile chemistry and processing flexibility, epoxy resins are widely used as matrix materials in advanced composite applications. They are valued for their excellent adhesion, high strength, low curing shrinkage, thermal stability, and good resistance to environmental degradation. In addition, epoxy matrices provide strong interfacial bonding with reinforcing fibres, ensuring efficient load transfer and improved composite performance. These properties make epoxy resins particularly suitable for high-performance applications in aerospace, automotive and other engineering industries [4].

The highly crosslinked architecture of cured epoxy resins is also the primary reason for their limited recyclability [4,20]. The three-dimensional network contains numerous ether linkages and aromatic structures, which provide excellent thermal and chemical stability. Consequently, degradation of the epoxy matrix requires sufficiently aggressive reaction conditions capable of cleaving covalent bonds within the polymer network. Understanding the chemical structure of the matrix is therefore essential for interpreting the effectiveness of the hydrolysis treatments investigated in this study.

**Figure 3:**
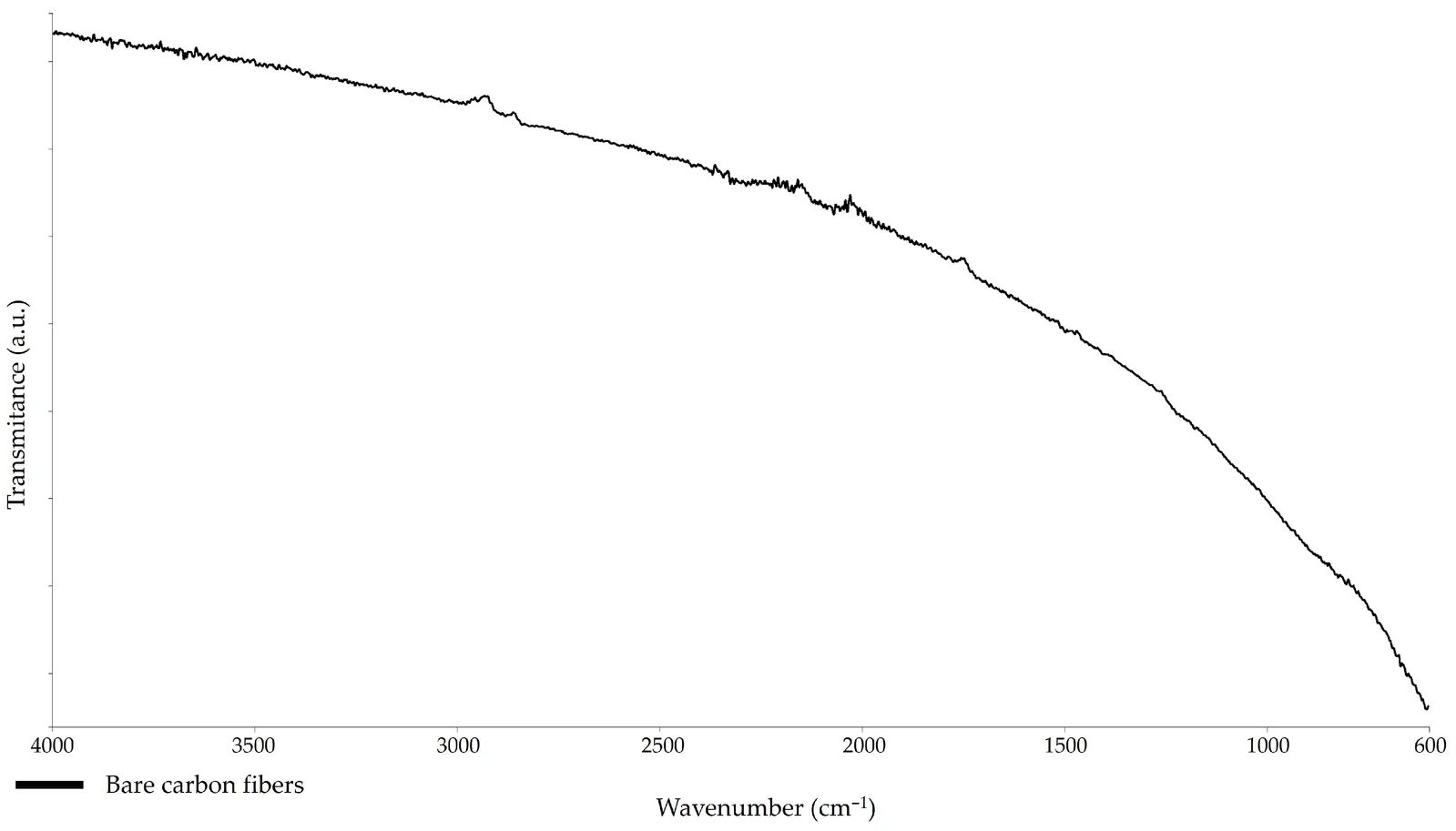
FTIR spectrum of bare carbon fibres.

The FTIR spectra (Figure 3) of bare carbon fibres show limited infrared activity due to the low concentration of polar functional groups. Consequently, the spectrum appears nearly featureless in the investigated mid-infrared region.

**Figure 4:**
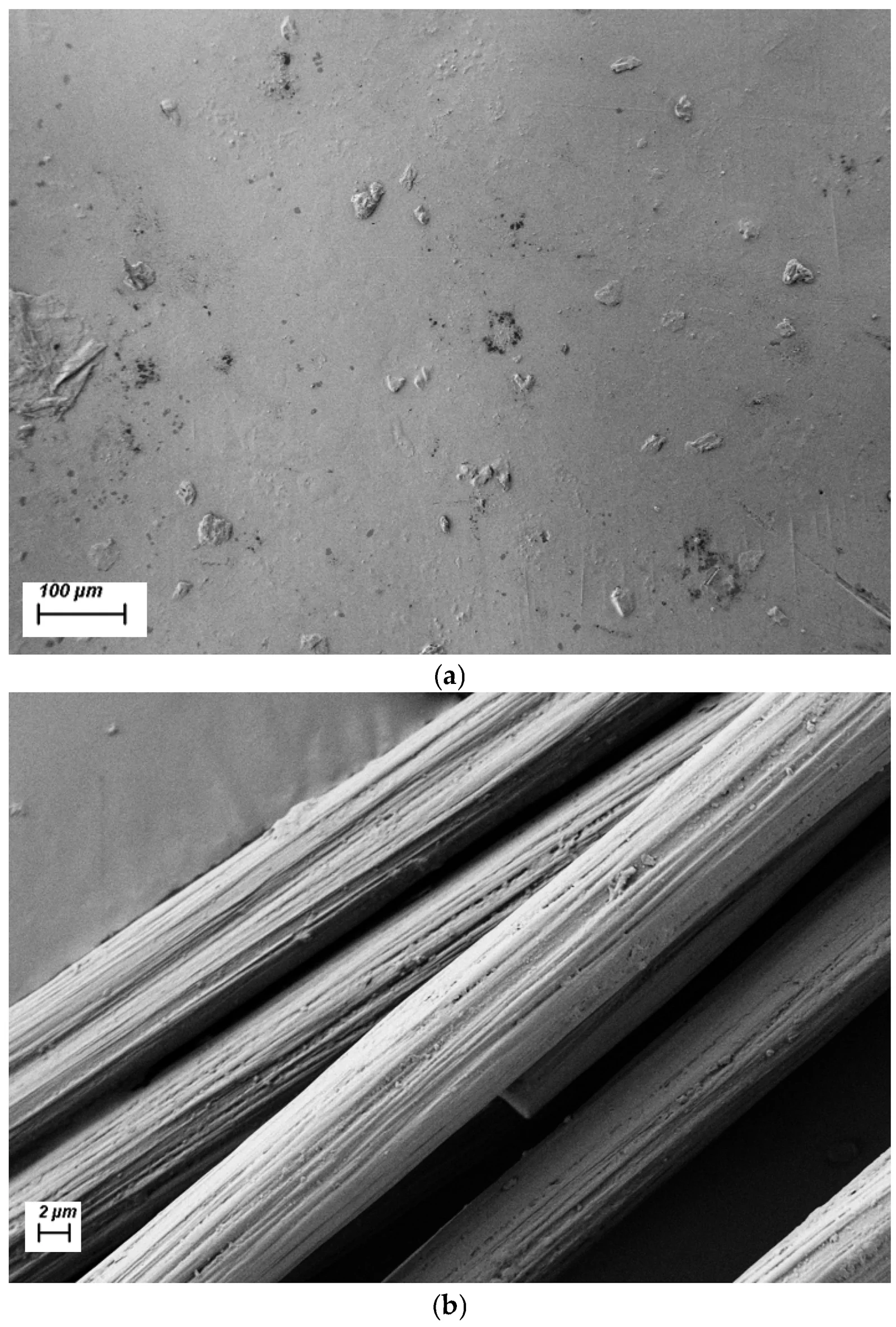
SEM images CFRP samples (**a**) and bare carbon fibres (**b**).

The SEM image (Figure 4a) of the CFRP composite shows a relatively homogeneous surface morphology. The polymer matrix appears mostly continuous, completely enveloping and almost entirely concealing carbon fibres within its bulk structure, with present small fragments likely originating from reinforcement or filler residues. The surface displays a rough and irregular texture typical of fractured or mechanically processed composite materials. In contrast the SEM image of the bare carbon fibres (Figure 4b) reveals well-defined, highly oriented fibres with a uniform cylindrical shape. The fibre surfaces are relatively smooth, with characteristic longitudinal grooves resulting from the manufacturing process. These fibres exhibit good structural integrity, which is essential for effective load transfer in carbon fibre reinforced composites.

### 3.2. Neutral Hydrolysis

**Figure 5:**
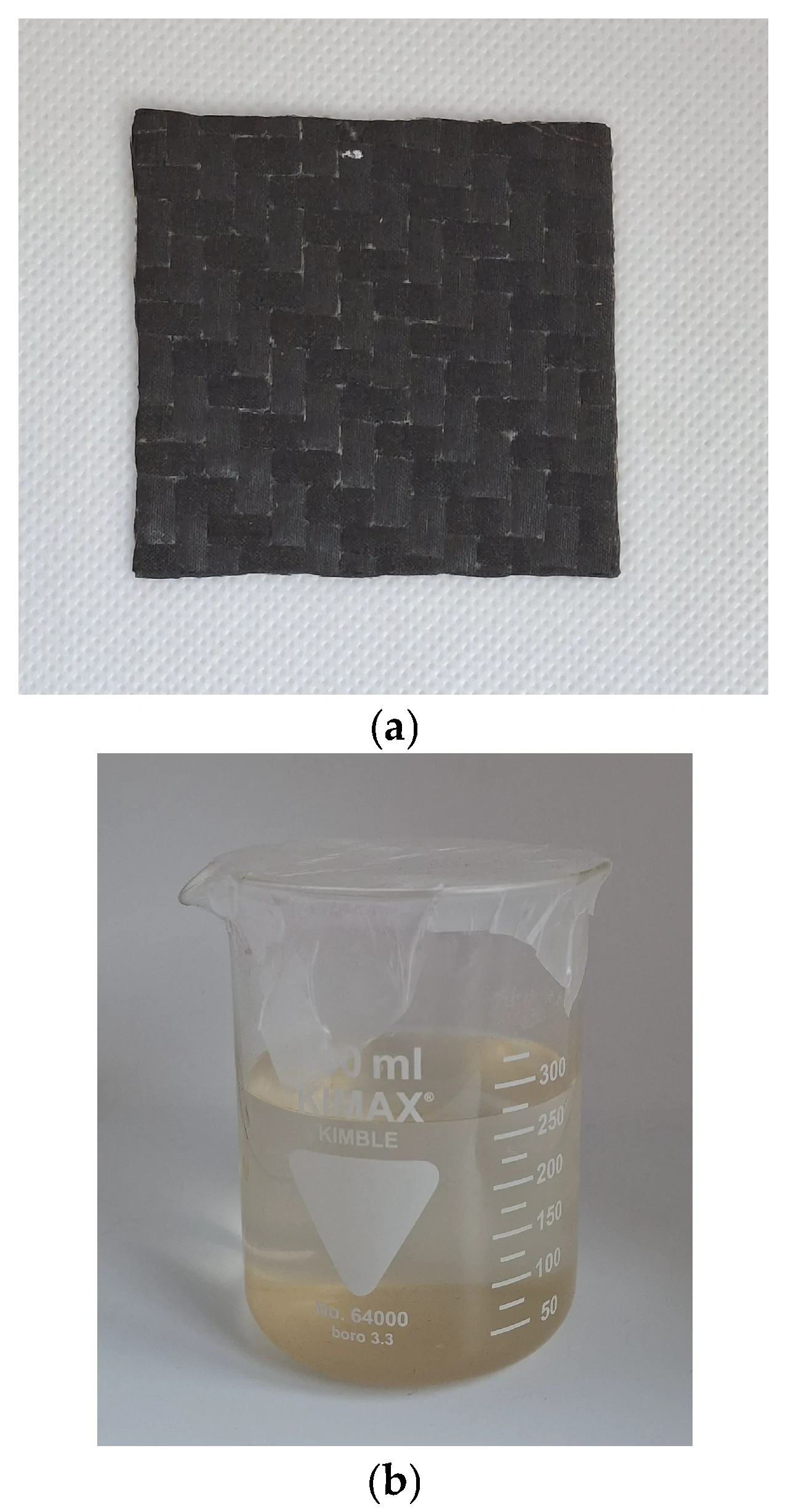
Products after neutral hydrolysis for sample 1: (**a**) sample 1 after neutral hydrolysis; (**b**) liquid residual.

Neutral hydrolysis was performed on sample 1. The temperature and pressure profiles recorded during neutral hydrolysis are presented in Table 1. The initial mass of the input sample was 1.5975 g. After the reaction conditions were reached (250 °C and 40 bar, approximately 60 min), the reaction was continued for a further 60 min. Subsequent visual inspection showed that the sample remained largely unchanged, indicating that the hydrolysis process did not significantly degrade the polymer matrix (Figure 5).

The limited effectiveness of neutral hydrolysis can be attributed to the relatively low reactivity of water toward the highly crosslinked epoxy network. Although elevated temperature and pressure increase the diffusivity and reactivity of water molecules, cleavage of covalent bonds within the epoxy structure remains relatively slow in the absence of catalytic species. As a result, only limited matrix degradation of the matrix can be expected under the applied conditions [1,5,21].

However, subsequent gravimetric analysis revealed a slight decrease in mass to 1.5251 g. In addition, the liquid phase showed a faint yellow discolouration, indicating that a chemical transformation had taken place during the process.

During neutral hydrolysis, water molecules may react with hydrolytically susceptible groups within the epoxy network, resulting in limited chain scission and the formation of small amounts of low-molecular-weight degradation products. Measurements of the pH and conductivity of the liquid residue after hydrolysis showed that the pH increased from 7.456 to 8.336, indicating that the solution became slightly more alkaline. In addition, the conductivity increased from 1.048 to 135.6 μS/cm, suggesting the presence of a higher concentration of dissolved ionic or ionisable species. These observations indicate that a limited amount of degradation products was released into the solution during hydrolysis. Nevertheless, the overall decomposition of the epoxy matrix remained very low, demonstrating that neutral hydrolysis was largely ineffective. Therefore, filtration of the liquid phase was not considered necessary.

**Figure 6:**
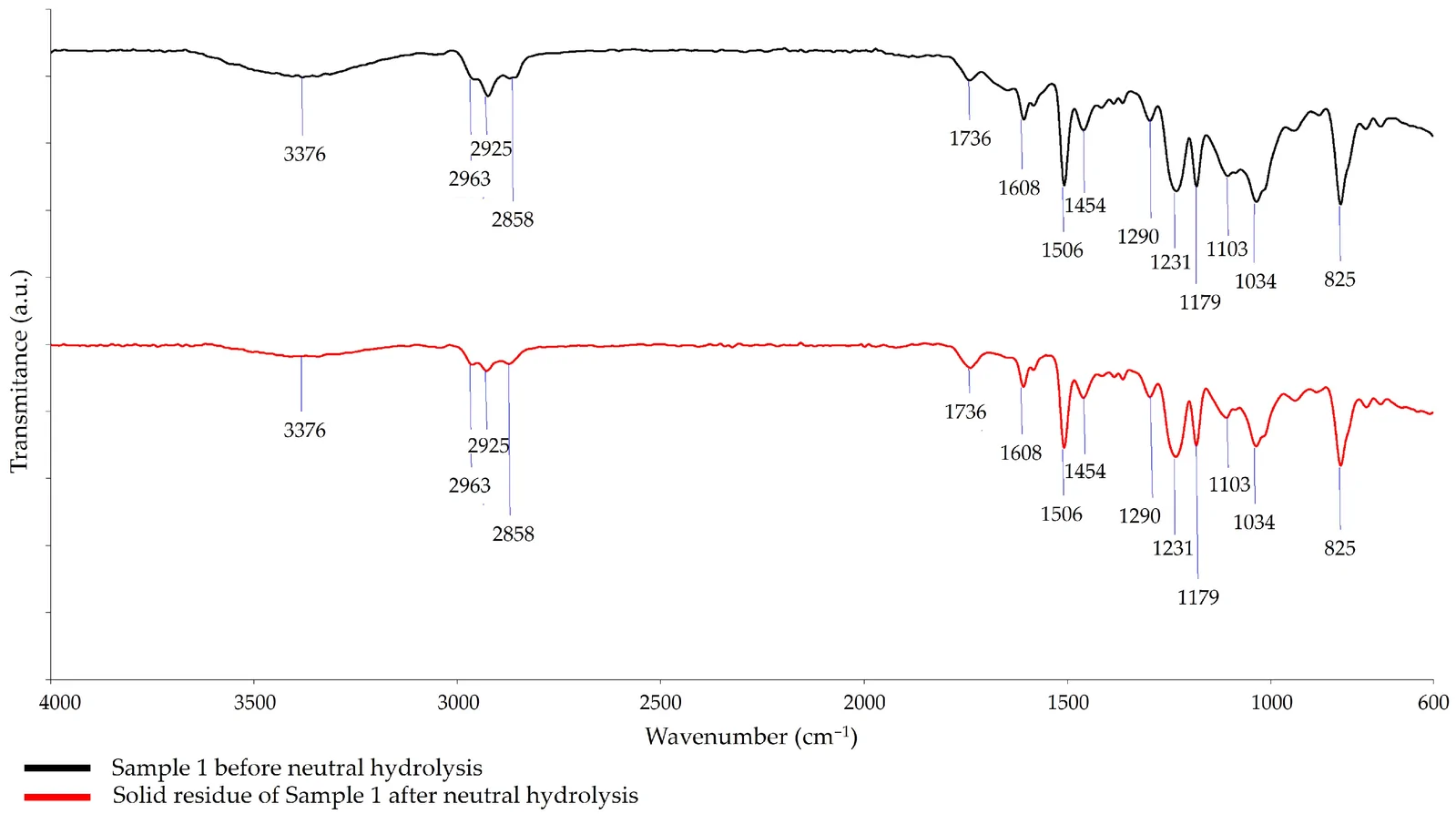
FTIR spectra of sample 1 before and after the neutral hydrolysis.

Analysis of the FTIR spectra indicates that the sample 1 remained largely unchanged after neutral hydrolysis. Although minor shifts in the signals were observed, they did not indicate any significant structural alterations (Figure 6). Therefore, it can be concluded from the spectra that the hydrolysis was ineffective, and chemical recycling of the starting material was not achieved.

The persistence of the characteristic epoxy signals indicates that the polymer network remained largely intact after treatment. Although hydrolysis may have induced localised bond cleavage, the extent of degradation was insufficient to substantially alter the overall chemical composition of the composite. These observations are consistent with previous studies reporting limited degradation of epoxy-based CFRPs during non-catalytic hydrolysis [1,5].

**Figure 7:**
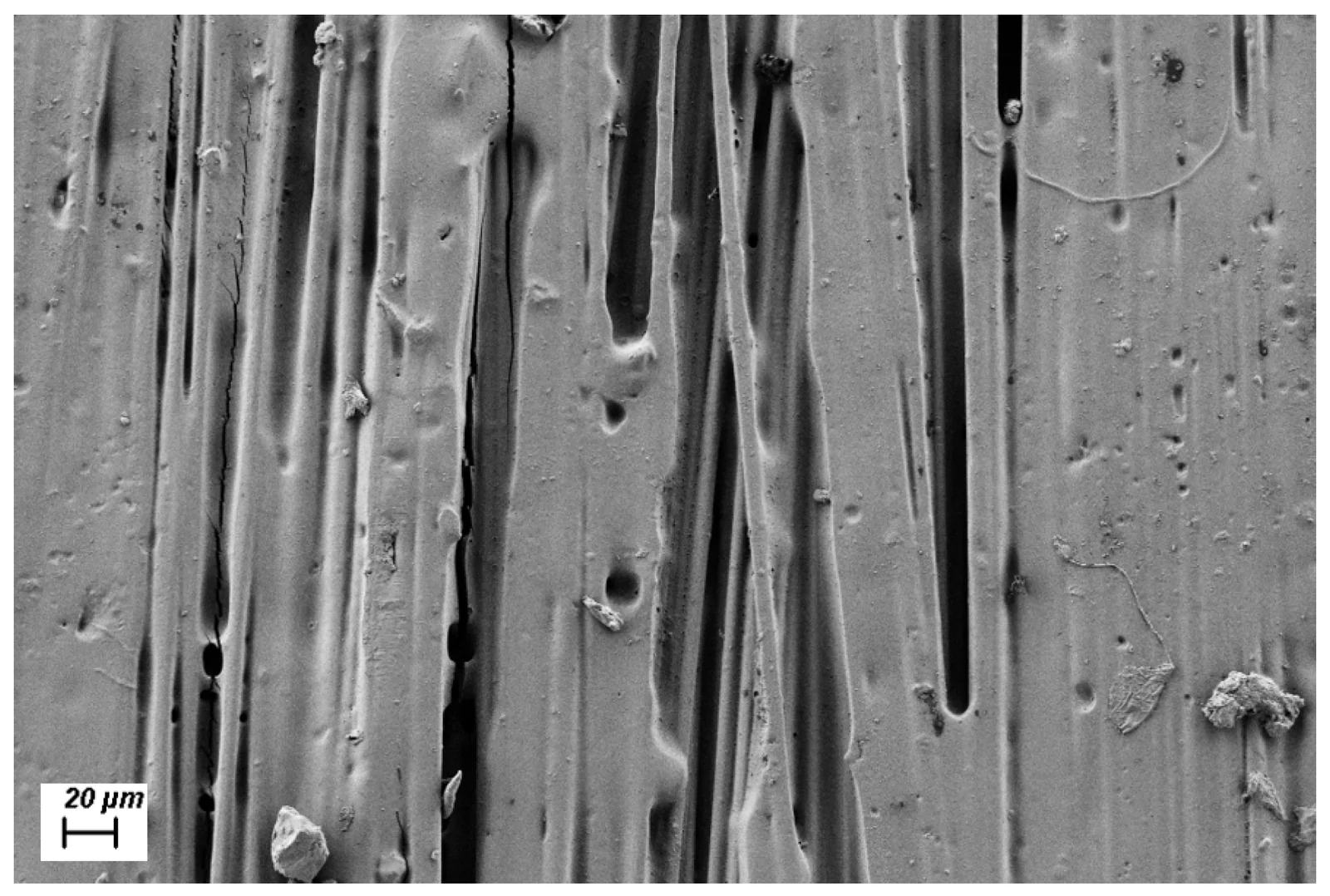
SEM image of sample 1 after the neutral hydrolysis.

Although FTIR spectroscopy detected no significant chemical changes, SEM analysis revealed clear morphological changes in the CFRP sample after neutral hydrolysis. The SEM image (Figure 7) shows partial degradation and removal of the epoxy-based polymer matrix, leading to the formation of elongated grooves, voids, and surface irregularities along the fibre direction. These observations indicate that neutral hydrolysis induced localised degradation of the polymer matrix at the microstructural level, even though such changes were not detectable by FTIR spectroscopy.

The apparent discrepancy between FTIR and SEM observations highlights the importance of employing complementary characterisation techniques when evaluating CFRP recycling processes. While FTIR provides information on the overall chemical composition of the material, SEM is considerably more sensitive to localised surface morphological modifications and partial matrix removal. Consequently, limited microstructural degradation may be detected by SEM even when the overall chemical signature of the composite remains largely unchanged.

**Figure 8:**
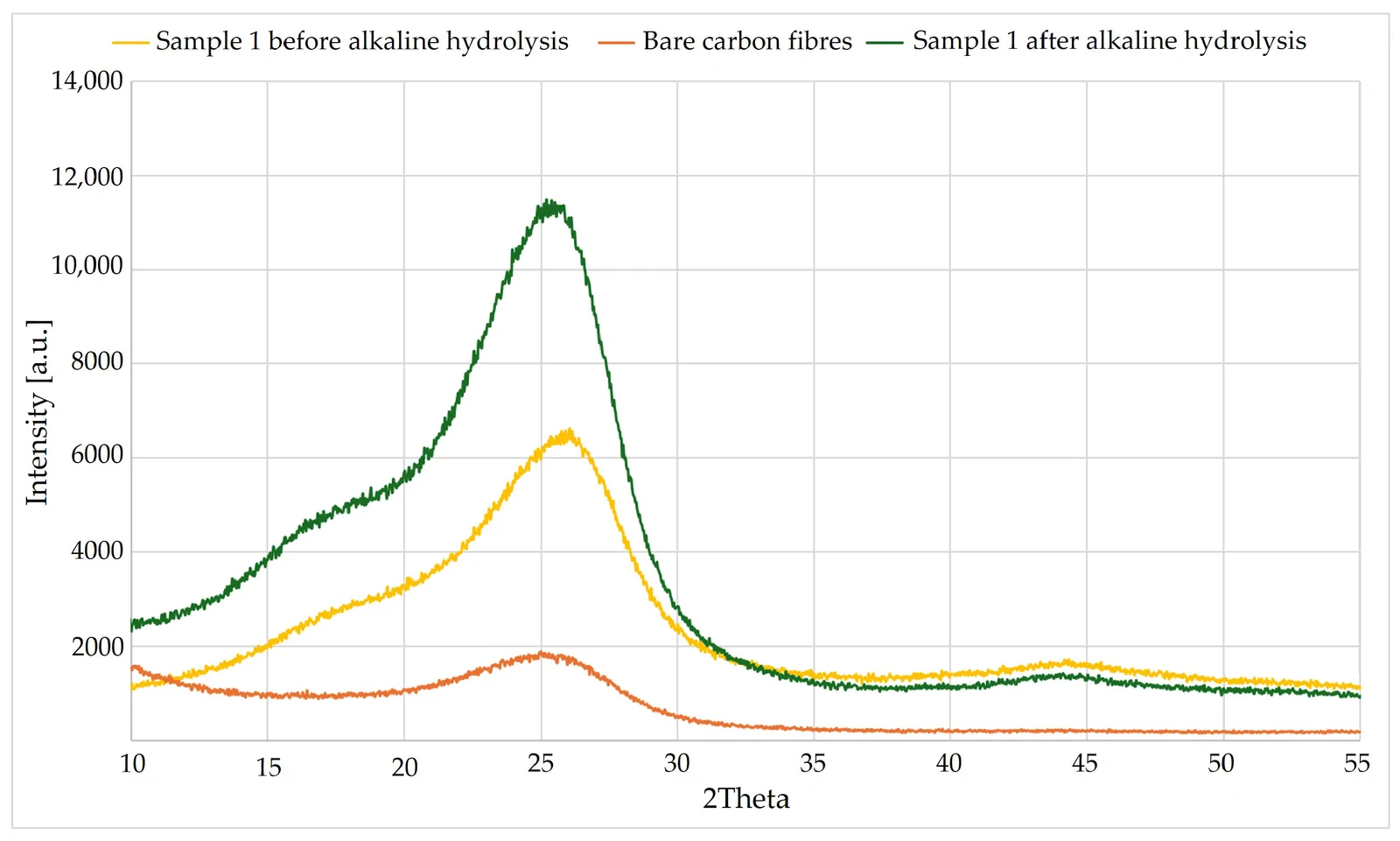
XRD patterns of sample 1 before and after the neutral hydrolysis and bare carbon fibres.

Figure 8 presents the X-ray diffraction (XRD) patterns of Sample 1 before neutral hydrolysis and recovered material after neutral hydrolysis, and the reference bare carbon fibres. All samples exhibit a broad diffraction peak centred at approximately 2θ = 25–26° (indicative of the (002) crystallographic plane, arising from presence of graphite crystallites) [22], while both epoxy-containing samples also show a shoulder-like feature in 2θ = 15–20° range. The similarity between the diffraction patterns of the untreated CFRP sample and the material recovered after neutral hydrolysis suggests that complete matrix degradation was not achieved. The persistence of broad diffraction features characteristic of the composite material indicates the presence of residual amorphous polymeric components after treatment.

### 3.3. Alkaline Hydrolysis

**Figure 9:**
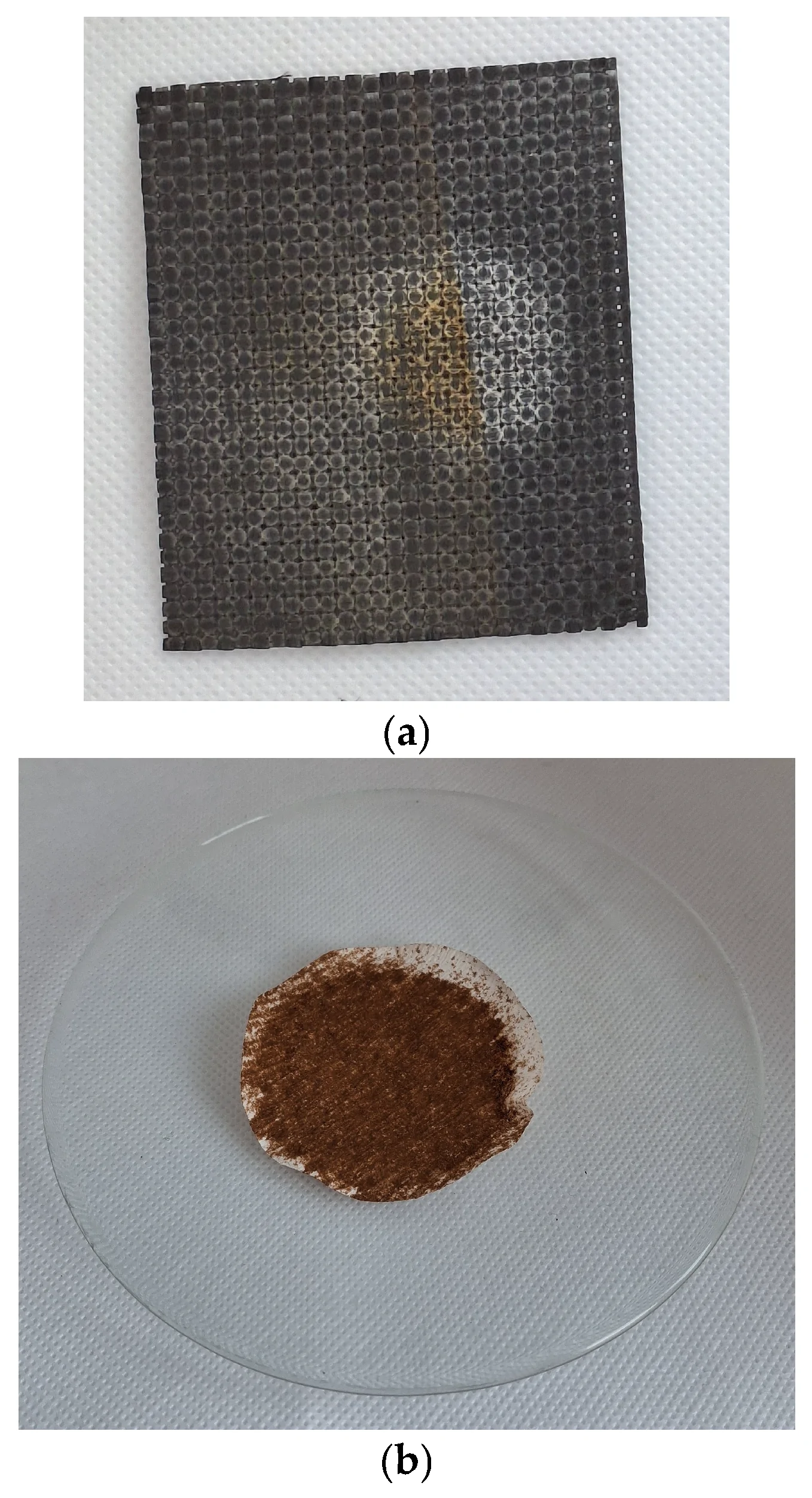
Product of alkaline hydrolysis for sample 2: (**a**) sample 2 after alkaline hydrolysis; (**b**) retentate after filtrate of liquid phase.

Two alkaline hydrolysis experiments (sample 2 and sample 3) were performed using different treatment durations, and their pressure and temperature profiles are presented in Table 2. In the first, a 1.1577 g input sample 2 of the composite material was used. After reaching the reaction conditions (250 °C and 40 bar, approximately 60 min), the reaction was continued for a further 60 min. The sample remained a piece of composite material, but the liquid phase had become darker in colour. The mass of the solid phase was 0.7616 g, indicating that partial depolymerisation of the polymer matrix had occurred, although complete matrix removal was not achieved (Figure 9a).

The pH of the liquid phase before the reaction (1.04 M NaOH solution) was 13.82, while the pH after hydrolysis reached 14.00. The highly alkaline conditions were therefore maintained throughout the treatment. The conductivity increased from 132 to 147.4 mS/cm, indicating the presence of additional dissolved ionic species in the liquid phase after hydrolysis. This change may be associated with the release of ionisable degradation products from the epoxy matrix. Under strongly alkaline conditions, these products may include phenolate species formed by deprotonation of phenolic groups generated during epoxy degradation.

This increase may be attributed to the release of degradation products from the epoxy matrix, including low-molecular-weight ionisable compounds formed through alkaline cleavage of susceptible bonds within the polymer network. Under strongly alkaline conditions, phenolic groups generated during epoxy degradation may undergo deprotonation to form phenolate ions, which could contribute to the observed increase in conductivity. The observed changes in pH and conductivity, together with the subsequent FTIR and SEM results, are consistent with partial degradation of the epoxy matrix during alkaline hydrolysis.

Under strongly alkaline conditions, hydroxide ions can promote the cleavage of susceptible bonds within the epoxy network, resulting in the formation of lower-molecular-weight degradation products that may dissolve in the aqueous phase. Consequently, alkaline hydrolysis is expected to achieve substantially greater matrix degradation than neutral hydrolysis performed under identical temperature and pressure conditions [9,16,23].

At this stage, the liquid phase was filtered, resulting in separation into a filtrate and a retentate. The retentate was then dried on filter paper (Figure 9b), after which the FTIR spectrum of the residual solid phase from the composite was recorded.

**Figure 10:**
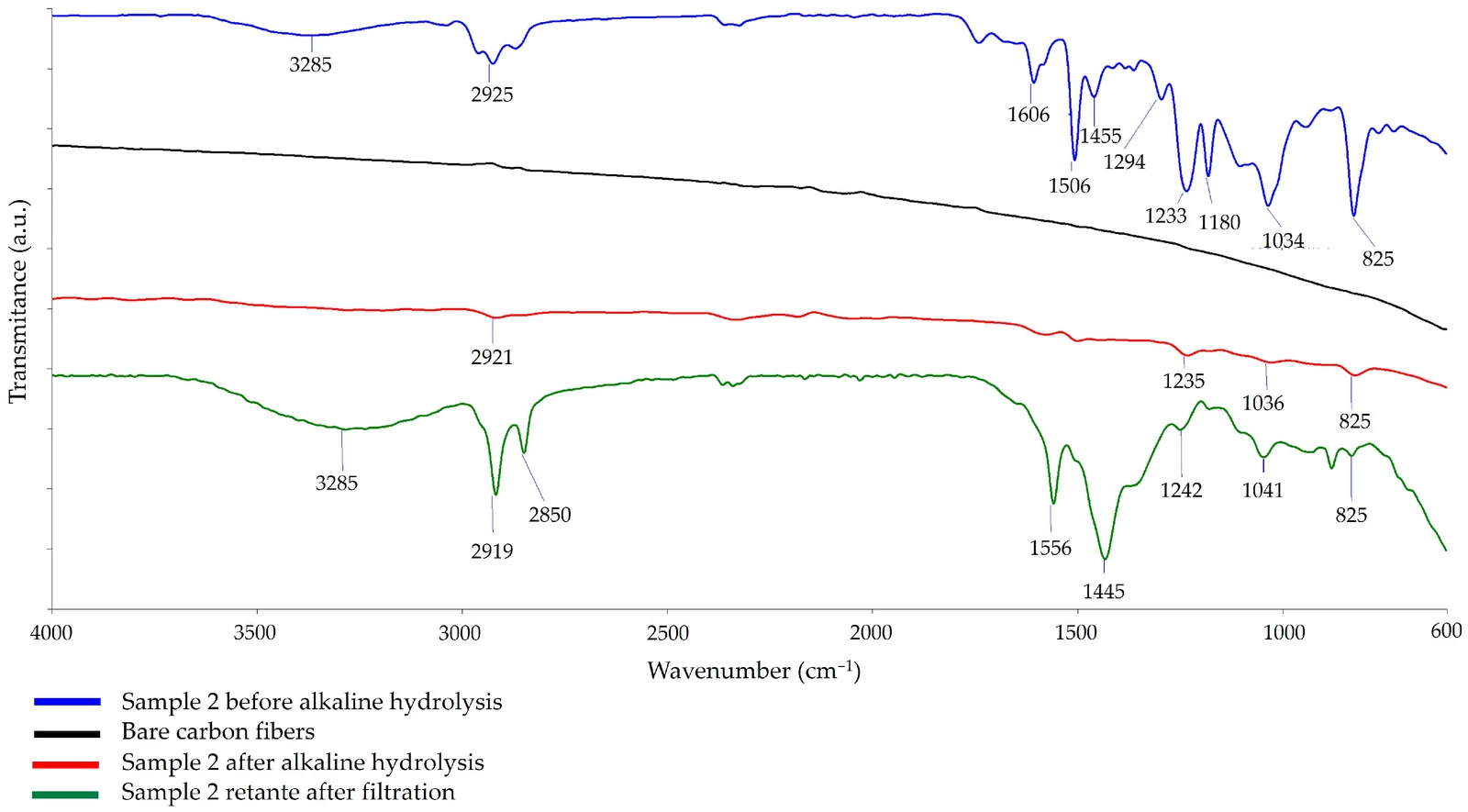
FTIR spectra of sample 2 before and after alkaline hydrolysis, bare carbon fibres and retentate recovered after filtrate.

As can be seen in Figure 10, the FTIR spectrum of the sample after alkaline hydrolysis is very similar to that of bare carbon fibres, the signals of the polymer matrix appear with very low intensity. This indicates that the chemical recycling of the starting material was largely successful, as the solid phase spectrum after the reaction almost completely matches that of the bare carbon fibres—an indication that the polymer matrix was effectively removed, which is the intended goal. The significant reduction in intensity of the characteristic epoxy signals indicates extensive degradation of the polymer matrix. Nevertheless, spectroscopic similarity to the reference carbon fibres does not necessarily imply complete matrix removal, as thin residual polymer layers may remain below the detection sensitivity of FTIR spectroscopy.

When the spectrum of the retentate of sample 2 after filtration was examined, it closely matched that of the original sample before alkaline hydrolysis, but some of signals are more intense. Considering the conditions of hydrolysis, it can be assumed that the polymer matrix dissolved in the NaOH solution at elevated temperature and the spectral differences—especially the shift of certain signals—are due to the degradation of the bonds between the carbon fibres and the surrounding polymer.

**Figure 11:**
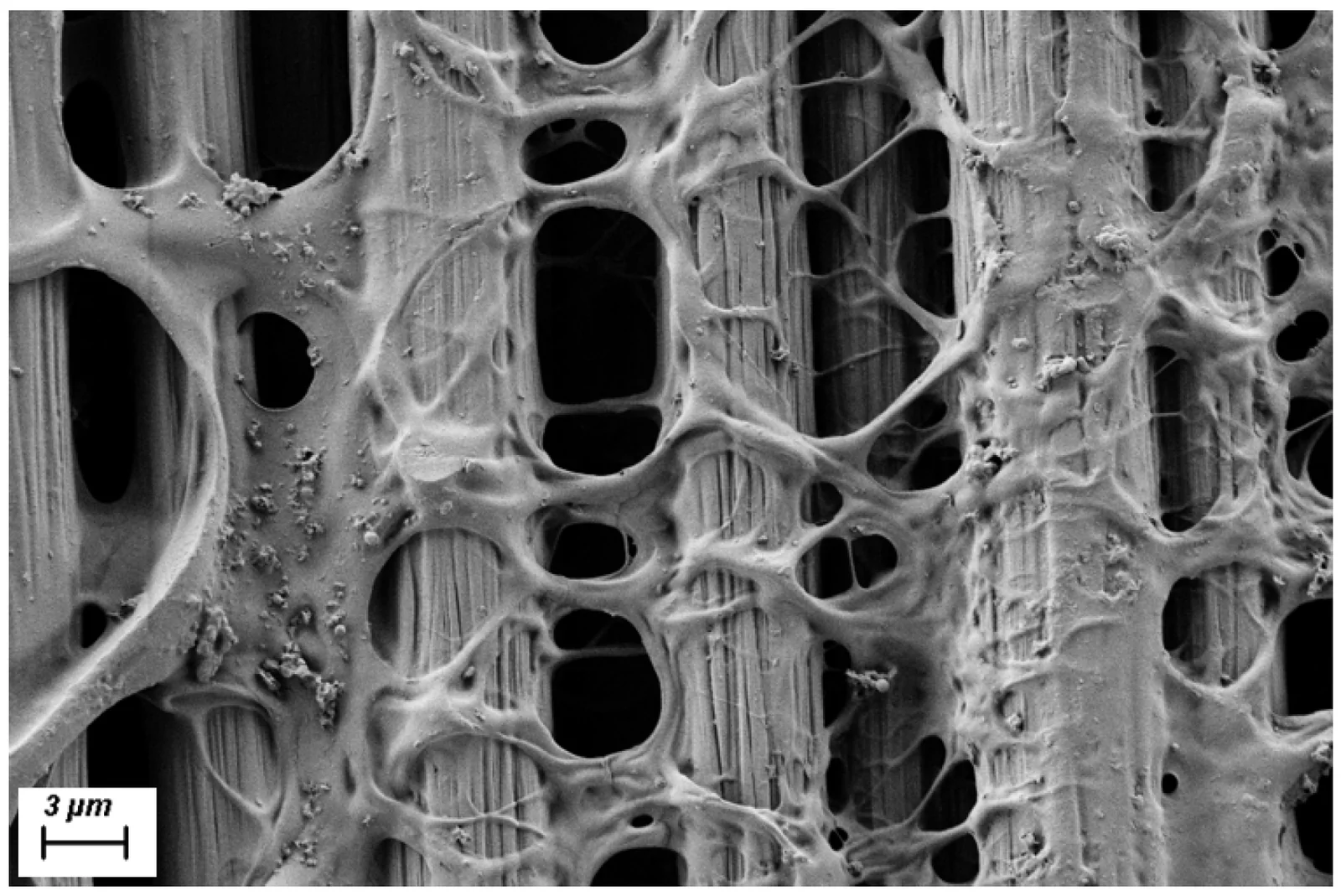
SEM image of sample 2 after the alkaline hydrolysis.

Although the FTIR spectrum of the sample after alkaline hydrolysis closely resembles that of bare carbon fibres, the SEM image of sample 2 after the alkaline hydrolysis (Figure 11) clearly shows that the epoxy-based polymer matrix remains present on the carbon fibres. The SEM micrograph reveals carbon fibres partially covered by residual epoxy, indicating incomplete removal of the polymer matrix. The surface appears heterogeneous, with areas where the matrix remains adhered to the fibres, forming patches and irregular surface features. These observations suggest that, despite the FTIR results indicating effective matrix degradation, alkaline hydrolysis did not achieve complete removal of the epoxy matrix at the microstructural level.

The combined FTIR, SEM, and XRD results demonstrate that relying on a single characterisation technique may lead to an overestimation of recycling efficiency. While FTIR suggested nearly complete removal of the epoxy matrix, SEM observations revealed residual polymer coverage on the fibre surfaces, and XRD patterns still exhibited characteristics associated with the composite structure. This finding highlights the importance of multi-technique characterisation for the accurate assessment of CFRP recycling performance [3,9].

**Figure 12:**
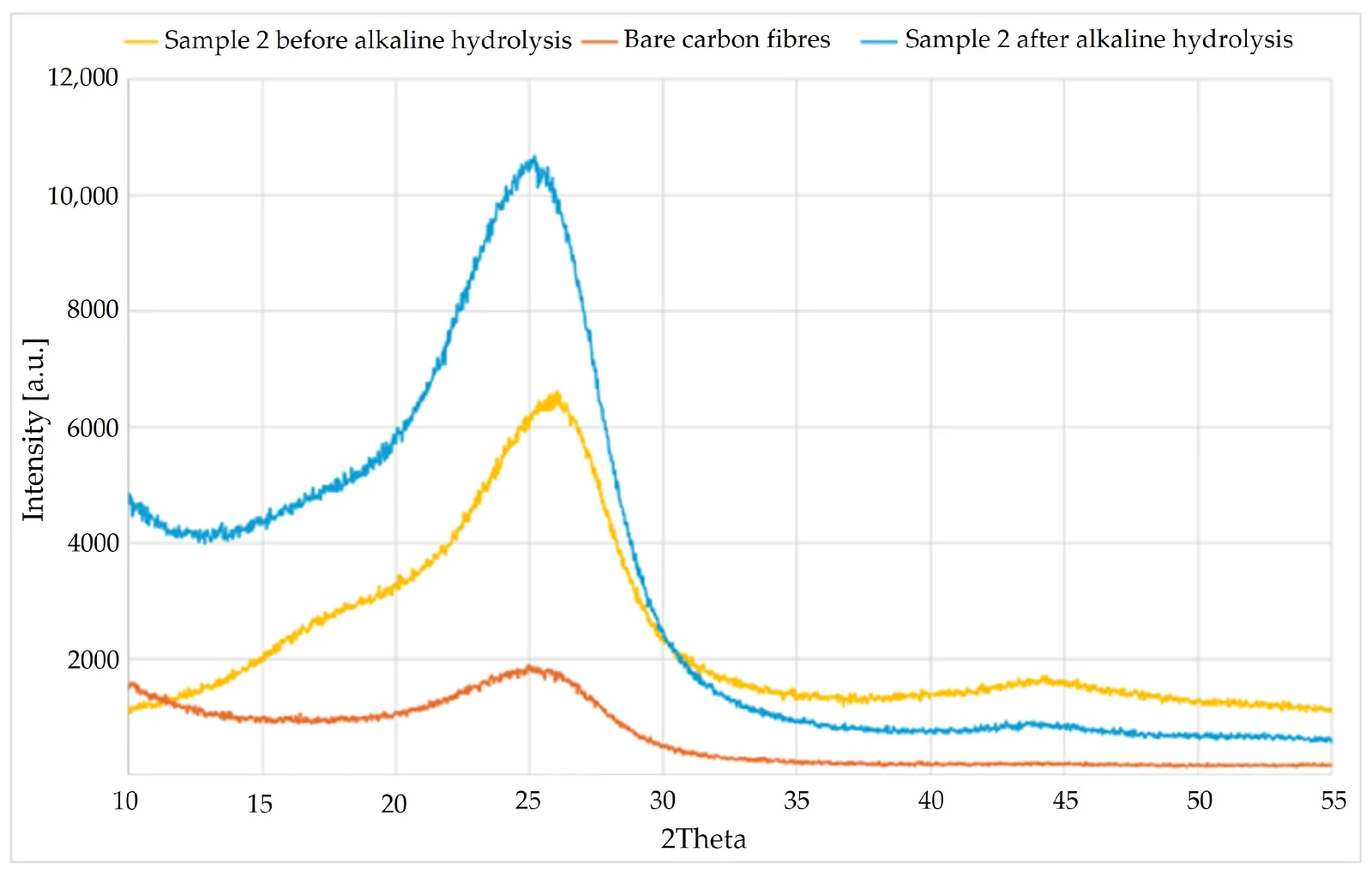
XRD patterns of sample 2 before and after the alkaline hydrolysis and bare carbon fibres.

Due to the incomplete alkaline hydrolysis observed under the applied reaction conditions (60 min after reaching 250 °C and 40 bar), a second alkaline hydrolysis experiment was conducted with a reaction time of 120 min after reaching the reaction conditions (250 °C and 40 bar, approximately 60 min). Although sample 3 had a larger mass and laminate thickness than sample 2, extensive matrix removal and carbon fibre recovery were observed after 120 min of alkaline hydrolysis (Figure 12). The weight of the input sample was 9.6146 g, while the solid phase of the output sample weighed 5.8903 g. 

The pH of the liquid phases before and after the reaction was exactly the same, but the conductivity decreased from 132 to 126.4 mS/cm. The observed change in conductivity suggests a change in the ionic composition of the liquid phase during prolonged alkaline hydrolysis and may be associated with the formation and transformation of epoxy matrix degradation products.

This liquid phase, collected directly from the reactor, exhibited the darkest coloration among all samples. It was subsequently filtered and weighed.

The XRD patterns of the untreated and hydrolysed CFRP samples exhibit a broad diffraction peak centred at approximately 2θ = 26°, which is characteristic of carbon fibre-reinforced composites. Following alkaline hydrolysis, the diffraction pattern remains similar to that of the untreated sample, indicating that a significant portion of the composite structure was retained. The persistence of broad amorphous features suggests that the epoxy matrix was not completely degraded or removed during the treatment. Therefore, the XRD results confirm that alkaline hydrolysis for 60 min was only partially effective in removing the epoxy matrix.

**Figure 13:**
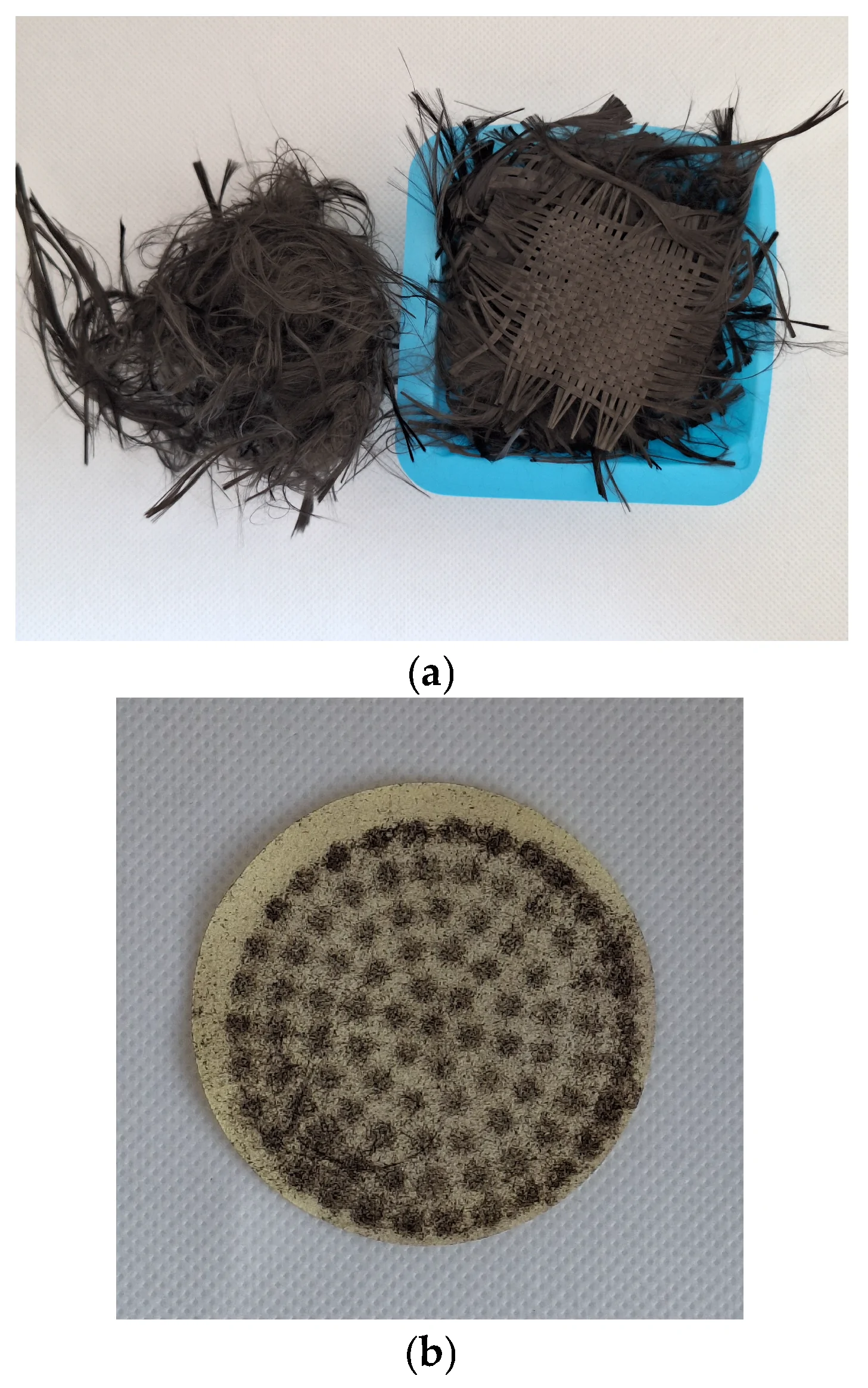
Product of alkaline hydrolysis for sample 3: (**a**) sample 3 after alkaline hydrolysis; (**b**) filter with the retentate.

The final mass of the retentate after alkaline hydrolysis of sample 3 was 0.0134 g, indicating that most of the degraded polymer matrix remained dissolved or dispersed in the filtrate. FTIR spectra were subsequently recorded for the recovered solid phase (Figure 13a) and the retentate (Figure 13b).

**Figure 14:**
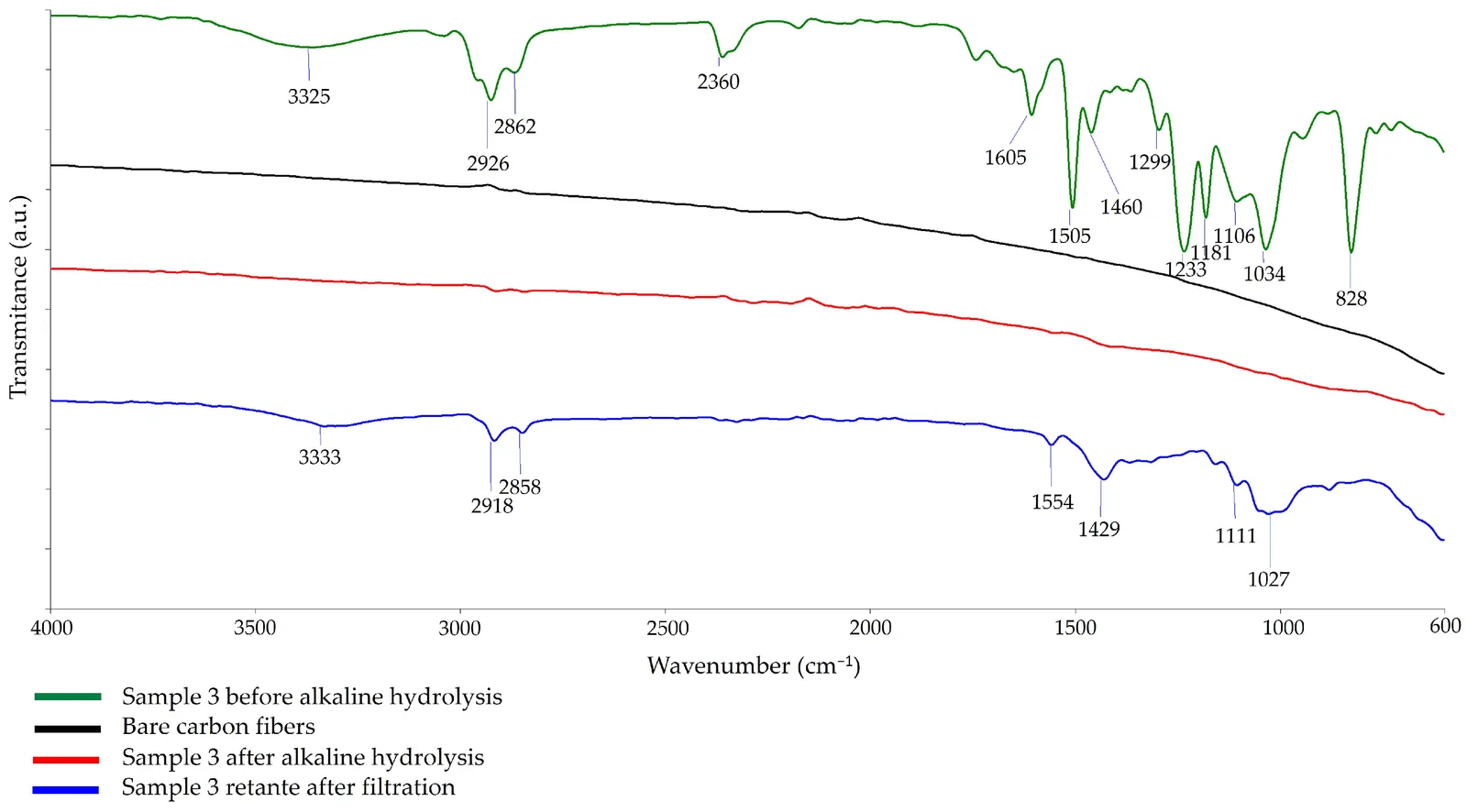
FTIR spectra of sample 3 before and after alkaline hydrolysis, bare carbon fibres and retentate after filtrate of liquid phase.

As shown in Figure 14, the FTIR spectrum of the recovered solid phase (sample 3 after alkaline hydrolysis) obtained after 120 min of alkaline hydrolysis closely matches that of the bare carbon fibres. The FTIR spectrum of the retentate after prolonged alkaline hydrolysis matches that of the retentate obtained under the previous reaction conditions. Some peak shifts are clearly visible, but we can assume that they are a consequence of the missing bonds connecting both materials of our composite.

The close agreement between the FTIR spectra of the recovered material and the reference carbon fibres suggests that prolonged alkaline treatment enabled nearly complete removal of the epoxy matrix. The absence of characteristic epoxy absorption bands indicates extensive depolymerisation and dissolution of the polymer network under the applied reaction conditions.

**Figure 15:**
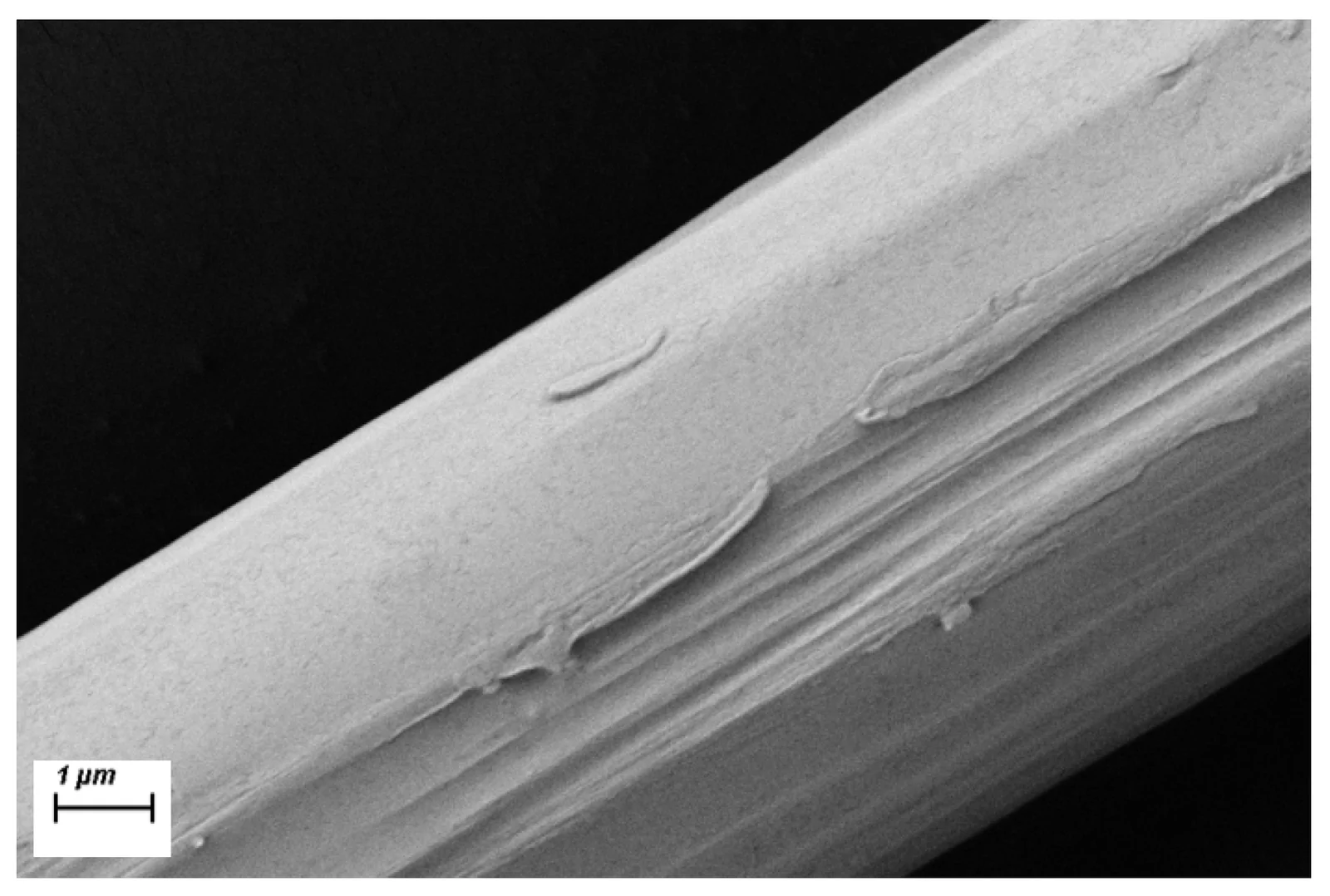
SEM image of sample 3 after the alkaline hydrolysis.

The SEM image of sample 3 after alkaline hydrolysis (Figure 15) shows a morphology closely resembling that of the bare carbon fibres (Figure 4b). The fibres remain well-defined and highly oriented, maintaining a uniform cylindrical shape with relatively smooth surfaces. The most effective alkali hydrolysis also does not affect the dimensions of the fibres; diameter of isolated fibres after epoxy removal is comparable to the bare carbon fibres. This indicates that prolonged alkaline hydrolysis effectively removed the polymer matrix without compromising the structural integrity of the fibres. This behaviour agrees with Jiang et al. [9], who reported that prolonged alkaline-assisted depolymerisation promotes more complete resin removal while preserving fibre integrity. Preservation of fibre morphology is essential for ensuring effective load transfer in carbon fibre reinforced composites and highlights the potential of CFRP recycling for the recovery of high-quality carbon fibres for reuse in new composite materials [24].

**Figure 16:**
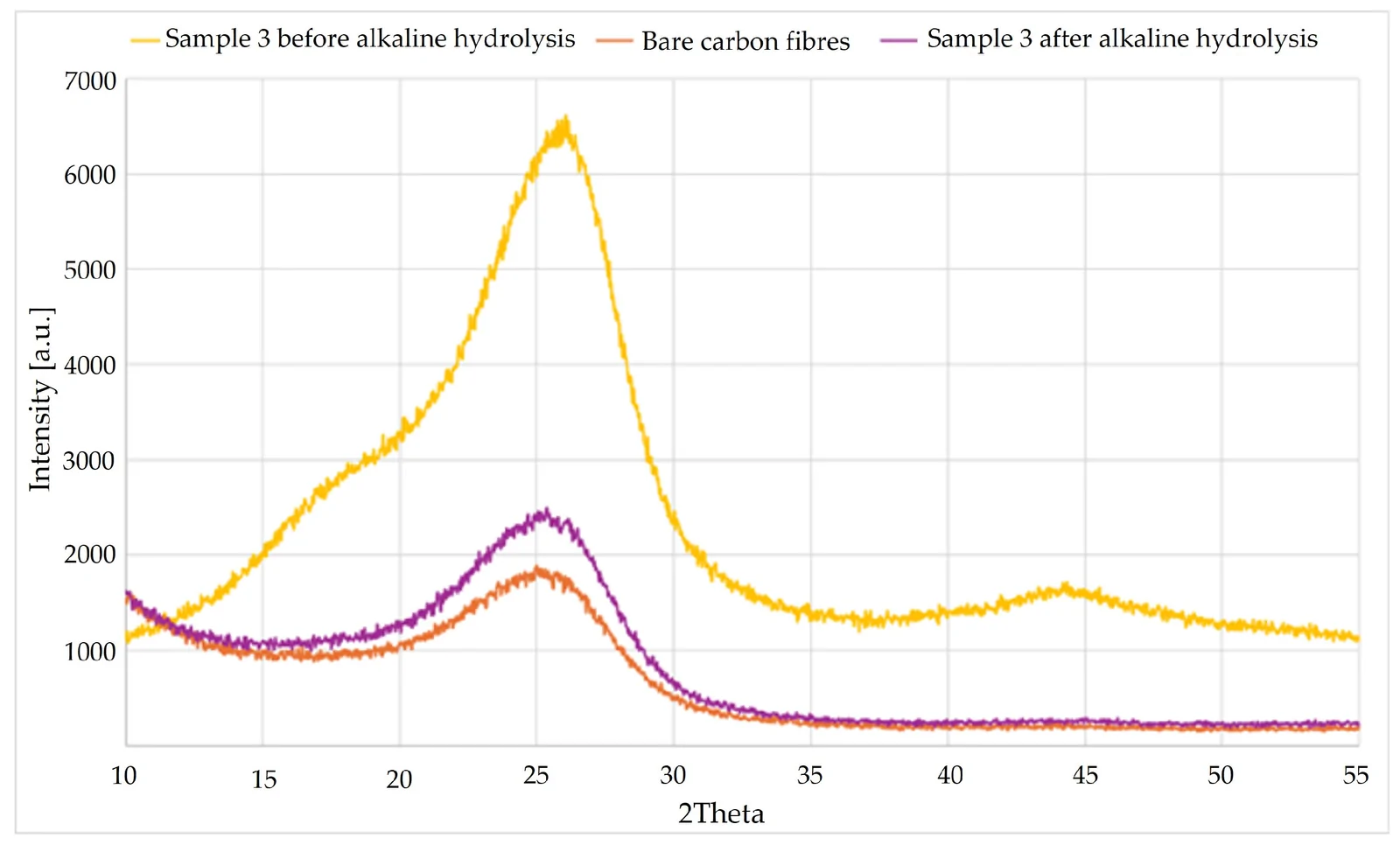
X-ray diffraction (XRD) patterns of sample 3 before and after the alkaline hydrolysis and bare carbon fibres.

Figure 16 presents the XRD patterns of Sample 3 before alkaline hydrolysis, the fibres recovered after alkaline hydrolysis, and the reference bare carbon fibres. All samples exhibit a characteristic diffraction peak at approximately 2θ = 25–26°.

Unlike the previous samples, the diffraction profile of the fibres recovered after prolonged alkaline hydrolysis closely resembles that of the reference bare carbon fibres and lacking other discerning features that arise from the presence of polymer, i.e., shoulder in the 2θ = 15–20° region. The similarity in peak shape and overall diffraction behaviour indicates that the prolonged alkaline hydrolysis treatment effectively removed most of the polymer matrix, resulting in a material with structural characteristics comparable to those of pristine carbon fibres.

Overall, the XRD results indicate that prolonged alkaline hydrolysis effectively removed the polymer matrix while preserving the characteristic crystalline structure of the recovered carbon fibres. Together with the SEM observations, these findings confirm that the applied treatment conditions enabled efficient fibre recovery without observable structural damage. [25] Similar preservation of fibre morphology following chemical removal of epoxy matrices has recently been reported for acetolysis-based recycling systems, where clean and mechanically viable carbon fibres were recovered after matrix depolymerisation [12].

As mentioned above, the untreated CFRP sample exhibits additional broader diffraction features associated with the composite structure and the presence of amorphous polymeric components. After prolonged alkaline hydrolysis, these features are significantly reduced, suggesting substantial matrix degradation and isolation of the carbon fibres. Overall, the XRD results indicate that alkaline hydrolysis of sample 3 was considerably more effective in removing the polymer matrix while preserving the characteristic crystalline structure of the carbon fibres [26].

### 3.4. Comparative Evaluation of Hydrolysis Efficiency

Table 3 summarises the effectiveness of the investigated hydrolysis treatments based on gravimetric analysis and the overall assessment of matrix degradation. Neutral hydrolysis resulted in only 4.5% mass loss, indicating limited degradation of the epoxy matrix under the applied conditions. In contrast, alkaline hydrolysis substantially increased matrix decomposition, leading to mass losses of 34.2% and 38.7% after 60 and 120 min of treatment, respectively. Although the difference in mass loss between the two alkaline treatments was relatively small, FTIR, SEM, and XRD analyses revealed a considerably higher degree of matrix removal after 120 min. This indicates that mass loss alone does not fully reflect the efficiency of CFRP recycling, particularly when residual polymer remains attached to the fibre surfaces. Therefore, gravimetric analysis should be interpreted together with spectroscopic, microscopic, and crystallographic evidence to obtain a reliable assessment of matrix degradation and carbon fibre recovery.

## 4. Conclusions

This study systematically evaluated the influence of hydrolysis medium and treatment duration on the chemical recycling of epoxy-based carbon fibre-reinforced polymers (CFRPs). The results demonstrated that both parameters play a decisive role in determining the extent of epoxy matrix degradation and the efficiency of carbon fibre recovery.

Neutral hydrolysis performed in deionised water at 250 °C resulted in only limited degradation of the epoxy matrix. Although minor mass loss and localised microstructural changes were observed, FTIR and XRD analyses showed that the recovered material retained characteristics similar to those of the untreated composite. SEM observations further confirmed the presence of residual polymeric material on the fibre surfaces, indicating that complete matrix removal was not achieved under the investigated conditions.

In contrast, alkaline hydrolysis in a 1.04 M NaOH solution substantially enhanced degradation of the epoxy network and promoted carbon fibre recovery. While treatment for 60 min resulted in significant matrix decomposition. However, SEM and XRD analyses revealed that residual polymer remained attached to the fibres. Alkaline hydrolysis resulted in substantially greater mass loss, reaching 34.2% after 60 min and 38.7% after 120 min. More extensive matrix removal and carbon fibre recovery were observed after the 120 min treatment. However, since the specimens used for the two alkaline treatments differed in mass and laminate thickness, the observed differences cannot be attributed exclusively to treatment duration. The recovered material exhibited FTIR spectra, surface morphology, and diffraction characteristics closely comparable to those of the reference carbon fibres, indicating preservation of fibre morphology and characteristic crystalline structure during the recycling process.

A key finding of this work is that the evaluation of CFRP recycling efficiency cannot rely on a single characterisation technique. The apparent discrepancy observed between FTIR, SEM, and XRD results after 60 min of alkaline hydrolysis demonstrated that spectroscopic evidence alone may overestimate the extent of matrix removal. The combined use of complementary spectroscopic, microscopic, and X-ray techniques therefore provides a more reliable assessment of recycling performance.

Overall, the results confirm that alkaline hydrolysis is considerably more effective than neutral hydrolysis for recycling epoxy-based CFRP waste under the investigated conditions. Furthermore, the study highlights the importance of treatment duration in achieving complete matrix degradation and recovery of structurally preserved carbon fibres. The results demonstrate that under identical temperature and pressure conditions, the reaction medium strongly influences matrix degradation efficiency. Although the study provides a direct comparison of neutral and alkaline hydrolysis under identical temperature and pressure conditions, further work involving repeated experiments, additional sample geometries, and mechanical testing of the recovered fibres would be valuable to confirm the broader applicability of the proposed approach. These findings contribute to the growing body of knowledge on chemical recycling of thermoset composites and demonstrate the potential of hydrolysis-based approaches for sustainable recovery of carbon fibres from end-of-life CFRP materials.

## Figures and Tables

**Table 1:** Monitoring of temperature and pressure over time for neutral hydrolysis.

Neutral Hydrolysis		
Sample 1		
Minute	p (bar)	T (°C)
10	1.2	106
20	3	143
30	5.9	162
40	16	200
50	32	235
60	40	250

**Table 2:** Monitoring of temperature and pressure over time for alkaline hydrolysis.

Alkaline Hydrolysis					
Sample 2			Sample 3		
Minute	p (bar)	T (°C)	Minute	p (bar)	T (°C)
10	1	94.5	10	0.5	81.7
20	2	127	20	2	131.5
30	3.8	148	30	4	151.1
40	10	186	40	18	209.8
50	23	221	50	36	246.8
60	37	246	60	39	250

**Table 3:** Comparative evaluation of hydrolysis treatments.

Sample	Medium	Time (min)	Mass Loss (%)	Matrix Degradation
Sample 1	Deionised water	60	4.5	Limited
Sample 2	1.04 M NaOH	60	34.2	Partial
Sample 3	1.04 M NaOH	120	38.7	Extensive

## Data Availability

Data can be provided by corresponding author on request.
